# Changes in positive and negative voice content in cognitive‐behavioural therapy for distressing voices

**DOI:** 10.1111/papt.12399

**Published:** 2022-05-06

**Authors:** Rachel M. Brand, Johanna C. Badcock, Georgie Paulik

**Affiliations:** ^1^ School of Health and Behavioural Sciences University of the Sunshine Coast Sippy Downs Queensland Australia; ^2^ Perth Voices Clinic Murdoch Western Australia Australia; ^3^ School of Psychological Science University of Western Australia Perth Western Australia Australia; ^4^ Discipline of Psychology Murdoch University Murdoch Western Australia Australia

**Keywords:** cognitive‐behavioural therapy, hallucinations, hearing voices, treatment

## Abstract

**Objective:**

People who experience distressing voices frequently report negative (e.g. abusive or threatening) voice content and this is a key driver of distress. There has also been recognition that positive (e.g. reassuring, or guiding) voice content contributes to better outcomes. Despite this, voice content has been neglected as a standalone outcome in evaluations of psychological therapies for distressing voices. We aimed to examine whether a modular cognitive‐behavioural therapy (CBT) intervention for voices led to changes in negative and positive voice content.

**Design/Methods:**

In a naturalistic, uncontrolled pre‐ and post‐ service evaluation study, 32 clients at an outpatient psychology service for distressing voices received eight sessions of CBT for distressing voices and completed self‐report measures of negative and positive voice content at pre‐, mid‐ and post‐ therapy.

**Results:**

There was no significant change in positive voice content. There was no significant change in negative voice content from pre‐ to post‐therapy; however, there was a significant change in negative voice content between mid and post‐treatment in which the cognitive therapy component was delivered. The CBT treatment was also associated with significant changes in routinely reported outcomes of voice‐related distress and voice severity.

**Conclusions:**

The cognitive component of CBT for distressing voices may be associated with changes in negative, but not positive, voice content. There may be benefit to enhancing these effects by developing treatments targeting specific processes involved in negative and positive voice content and further exploring efficacy in well‐powered, controlled trials with more comprehensive measures of voice content.

**Practitioner points**

- There is renewed recognition of the important role of voice content (i.e. what voices say) as a driver of distress related to voice hearing experiences.
- Current best practice CBT for distressing voices, with modules that include coping strategy enhancement and cognitive therapy may reduce negative voice content, but does not appear to change positive voice content.
- Modular CBT treatments may have some utility in changing negative voice content where this is a goal of the client, but further development and evaluation of these interventions is required.
- Practitioners are encouraged to assess not only voice frequency and distress when evaluating their therapies for distressing voices, but also negative voice content.

## INTRODUCTION

The experience of hearing a voice or voices in the absence of a corresponding external stimulus (also known as auditory verbal hallucinations) occurs across a number of psychiatric diagnoses as well as in the general population (Johns et al., 2014). When this experience becomes persistent and distressing it can lead to significant impairment and a need for clinical care (Johns et al., 2014; Loberg et al., 2019).

One important determinant of distress and a need for care is what the voices say, or the *content* of the voices. Indeed, Daalman et al. (2011) found that the presence of negative voice content was the best predictor of whether people were a patient or non‐patient in a sample of voice hearers. Early cognitive‐behavioural theories of voice hearing recognized the importance of voice content in driving negative beliefs about voice intent and power (i.e. beliefs about malevolence and omnipotence), which subsequently influenced the distress experienced in relation to the voice (Chadwick & Birchwood, 1994). However, the development of cognitive‐behavioural theory and interventions following this early work shifted focus from the role of negative content to an almost exclusive focus on the role of beliefs and appraisals (e.g. Peters et al., 2012). More recently, there has been a call for renewed focus on negative voice content in both theory and intervention (Larøi et al., 2019).

Surveys exploring the phenomenology of voices in large samples have helped to shed light on the degree and nature of negative voice content in psychiatric populations. Nayani and David's (1996) study of 100 voice hearers with schizophrenia‐spectrum disorder diagnoses found that the majority reported experiencing voices with negative content, with critical, derogatory, persecutory, threatening and commanding content endorsed by roughly 70% of people. Similarly, McCarthy‐Jones et al. (2014) examined the phenomenology of voices in a sample of 199 people with voices and a psychiatric disorder and found high levels of endorsement of voices being persecutory (50%), abusive (61%), derogatory (67%), threatening (63%) and critical (65%). Negative voice content has also been reported in samples with posttraumatic stress disorder, bipolar disorder, borderline personality disorder, dissociative identity disorder and substance use disorders (Waters & Fernyhough, 2017).

It should be noted that there are some complexities in defining negative voice content. Objective analysis of the literal, decontextualized linguistic content of voices (i.e. the actual words voices say) confirms negative content in about a third of clinical voice hearers (de Boer et al., 2021). However, content rated as ‘neutral’ by objective ratters is often rated as negative by the person experiencing the voices (van der Gaag et al., 2003), suggesting that other factors, in addition to literal linguistic content, are involved in perceptions of the valence of voice content. It has been noted that the tone and interpersonal context of voice utterances can also be important in perceptions of the valence of voice content (Larøi et al., 2019). For example, a seemingly innocuous comment, such as ‘great work’ may be experienced as negative if the tone is threatening or mocking, or if the speaker is identified as having hostile intentions. Deamer and Wilkinson (2015) highlight the importance of the perceived communicative intention of voices, conceptualizing voices as hallucinated acts of communication rather than just hallucinated sounds.

Interestingly, around 40%–50% of voice hearers in psychiatric populations report hearing voices perceived as benevolent and with positive content in addition to voices with negative content (for example, endorsing hearing voices that were helpful, guiding and affirming; McCarthy‐Jones et al., 2014; Nayani & David, 1996). These findings indicate that positive and negative voice content often co‐occur.

There is currently limited research into factors that contribute to or influence the valence of voice content; however, several psychological variables have been theorized to be involved. There is a growing body of research that implicates experiences of childhood trauma and adversity in the presence of negative voice content (Rosen et al., 2018). A recent study found that people whose trauma experiences were characterized by significant childhood sexual, physical and emotional abuse reported higher amounts of negative voice content (as compared to those with emotion‐focused, or no trauma; Begemann et al., 2021). Important parallels between the type of traumatic events that people have experienced and the specific content of their voices have also been noted (Corstens & Longden, 2013; Hardy et al., 2005). Experiences of childhood trauma have a number of psychological impacts, which may play a role in negative voice content. Research has particularly begun to explore the role of insecure attachment (Berry et al., 2012), negative schematic beliefs (Scott et al., 2020) and hypervigilance to social threat (Garwood et al., 2015), with indication that these processes may play a role.

Cognitive‐behavioural therapy (CBT) represents current best practice in treating distressing voices, with trials showing that CBT has small, but significant effects on voice hearing severity (van der Gaag et al., 2014). Cognitive‐behavioural approaches to voices generally involve a collaborative assessment and formulation of the factors contributing to a client's distress and an individualized intervention targeting these factors using a range of cognitive‐behavioural strategies. Interventions commonly include a focus on increasing coping and on addressing unhelpful beliefs about voices, which are empirically derived targets deemed to contribute to voice‐related distress. These components of CBT interventions may address some of the theorized mechanisms involved in negative voice content, such as negative schematic beliefs and hypervigilance to social threat and therefore impact upon negative voice content specifically. Treatment trials have focused on overall voice severity, frequency and distress (e.g. Badcock et al., 2020; Craig et al., 2018), or on compliance with voice commands, or beliefs about voice malevolence and omnipotence, as outcomes of interest (Birchwood et al., 2014). To date, we are not aware of any trials of psychological therapy for distressing voices that have included voice content as a primary outcome of interest. Given that negative voice content may be a key driver of voice‐related distress, there is growing consensus that negative voice content is a potentially important measure of treatment success (Larøi et al., 2019). Indeed, our clinical experience suggests that clients frequently come to psychological therapy with a hope of changing the negative content of their voices.

Given that positive voice content often co‐occurs with negative voice content, this is also a potentially important outcome. People who hear positive voice content have been found to relate this experience to a sense of companionship, protection, advice and personal growth (Valavanis et al., 2019). This potential relationship between positive voice content and aspects of wellbeing indicates that enhancing positive voice content could also be a fruitful aim of interventions. To date, this area has been overlooked in the evaluation of psychological interventions for voices.

## AIMS

In order to address the gaps in current knowledge regarding the impact of current psychological treatments for distressing voices on both negative and positive voice content, we analysed routine service evaluation data collected at [masked for blind review], a transdiagnostic outpatient psychological therapy service for distressing voices. We aimed to examine whether a modular CBT intervention with a focus on increasing coping and addressing unhelpful beliefs about voices led to changes in negative and positive voice content. To establish the general efficacy of the intervention, we also assessed changes in routinely reported outcomes of CBT for distressing voices (overall severity and distress).

## METHODS

### Study design

We examined the impact of a modular CBT treatment for distressing voices on both negative and positive voice content in a naturalistic, uncontrolled pre‐ and post‐ service evaluation study design.

### Participants

Participants were clients attending [masked for blind review] Clinic – an outpatient psychological therapy service for people who hear distressing voices, based in [masked for blind review]. The clinic takes a transdiagnostic, symptom‐focused approach and therefore provides treatment for people regardless of psychiatric diagnosis. The sole inclusion criteria for the clinic is that clients are experiencing distressing voices and are seeking psychological support for this. Exclusion criteria include: the presence of an acute phase of psychosis and/or high levels of thought disorder, active suicidal ideation or intent (fleeting suicidal ideation was accepted, with planning or intent to act as the cut off), or a significant history of violence towards others. These criteria are assessed by clinic clinicians in consultation with the client and with referrers.

### Procedure

Clients were offered eight therapy sessions in total (up to 1 h per session). The treatment offered at [masked for blind review] Clinic is described in more detail elsewhere ([masked for blind review] et al. 2019). In brief, treatment consists of two treatment modules; module one (four sessions) focuses on coping strategy enhancement (CSE; adapted from Tarrier et al., 1993, 1998) and module two (four sessions) focuses on cognitive therapy (taken from the ‘Guided self‐help cognitive‐behaviour Intervention for VoicEs’ (GiVE) programme (Hayward et al., 2012; Hazell et al., 2018). The CSE module aims to identify and reduce voice hearing triggers, improve coping and increase self‐esteem building activities in daily life. The cognitive therapy module aimed to re‐evaluate unhelpful beliefs about the self and voices, through identification of negative beliefs about the self and voices, and application of cognitive and behavioural techniques to support the re‐evaluation of these beliefs. Typical beliefs targeted in the self‐esteem module included ‘I am stupid’, ‘I am weak’ and ‘I am worthless’, with the therapist guiding the client to choose a negative belief that they hold, and the voice content also directly or indirectly feeds into. Typical beliefs targeted in the beliefs about voices module included ‘the voice has more control over my life than I do’, ‘the voice knows all’, ‘the voice can cause me/others harm’. The therapy is manualized but delivered with flexibility to individual needs and preferences.

Therapy was provided by two clinical psychologists, each with more than 15 years of experience in working psychologically with people experiencing psychosis, or one of 13 provisional psychologists (completing an accredited training program in clinical psychology) on placement at [masked for blind review] under the supervision of the senior author. The provisional psychologists delivering treatment attended 6 h of training in the intervention and attended weekly supervision.

Assessments took place at pretreatment (1 week prior to commencing therapy), mid‐ treatment (following the CSE module) and post‐treatment (following the cognitive module). Assessments were conducted using self‐report measures, which were completed by the client online using Qualtrics, either at their own home or in the clinic waiting room.

### Measures

*Demographic and clinical information* was gathered at pretreatment using a self‐report measure. Data was collected regarding age, gender, marital status, current employment, length of time since first hearing voices, and current medication. Information regarding current psychiatric diagnosis was taken from the client's referral letter from their GP.

*Negative voice content* was assessed via self‐report at pre, mid and post‐treatment using a sum of two items. The first item was taken from the Hamilton Program for Schizophrenia Voices Questionnaire (van Lieshout & Goldberg, 2007), a self‐report measure that asks respondents to rate aspects of their voices over the last week. The item used was ‘How bad are the things the voices say to you?’ (rated on a 0–4 Likert scale, from 0 = no voices saying bad things, to 4 = horrible). The second item was designed to assess the amount of voice content that the client perceived to be negative over the last week: ‘How often were your voices negative’ (rated on a 0–4 Likert scale, from 0 = never, to 4 = a lot).

*Positive voice content* was measured using a single item designed to assess the how often they perceived their voice content to be positive: ‘How often were your voices positive’ (rated on a 0–4 Likert scale, from 0 = never, to 4 = a lot).

*Overall voice severity* was measured using the Hamilton Program for Schizophrenia Voices Questionnaire (van Lieshout & Goldberg, 2007) severity factor identified by Berry et al. (2021). This includes six items and has been found to have good psychometric properties (Berry et al., 2021).

*Voice‐related distress* was measured using a single distress item from the Hamilton Program for Schizophrenia Voices Questionnaire (van Lieshout & Goldberg, 2007; ‘How distressing are the voices that you hear?’). This single item was chosen as a measure of voice distress, rather than the established distress factor (Berry et al., 2021) because the distress factor includes the negative voice content item and therefore may confound negative voice content with distress.

### Statistical analysis

Data analysis was conducted using SPSS, version 26.0. Non‐parametric analysis was considered most appropriate for the dependent variables measured on single or double item Likert scales (positive and negative voice content, voice‐related distress), as these were considered to be ordinal data (Jamieson, 2004). Differences in positive and negative voice content and voice‐related distress over the three time points were analysed using the Friedman test, a non‐parametric alternative to the repeated measures ANOVA. In the event of a significant omnibus test, post‐hoc analyses of differences between each timepoint were conducted using the Dunn‐Bonferroni's post‐hoc method. Kendall's W coefficient of concordance was used as a measure of effect size, yielding a figure between 0 and 1 that indicates the proportion of participants whose rank scores were in agreement (i.e. the proportion of participants who showed improvement in the expected direction). Kendall's W can be interpreted using Cohen's guidelines of 0.1 (small effect), 0.3 (moderate effect) and above 0.5 as a strong effect). Differences in overall voice severity over the three time points were analysed using a one‐way repeated measures ANOVA. Violations to normality in overall voice severity scores were corrected using square transformation and this statistical analysis conducted on the transformed data. Post‐hoc analyses of differences between each timepoint were conducted using a Bonferroni adjustment and partial eta squared was used as a measure of effect size.

## RESULTS

Data were collected between July 2019 and August 2021. During this time, 123 clients were offered therapy at [masked for blind review] Clinic. Of these, seven (5.7%) attended the initial assessment session but did not commence therapy and two (1.6%) did not consent to having their data used for service evaluation. Thirty‐five (28.5%) clients opted to do a different type of therapy for their voices (the other main treatment available at the clinic is imagery rescripting to treat underlying trauma). Of those who began treatment, six (4.9%) had not been in the service long enough to complete any follow‐up measures, 29 (23.5%) only completed the CSE module (with common reasons being that the client decided to commence imagery rescripting, the client had a social crisis that prevented them from continuing, the client was too thought disordered to conduct the cognitive module, or the client did not feel that they needed further therapy,) and six (4.9%) terminated therapy prior to completion of the cognitive module (reasons included a physical health crisis, clients commencing employment and two clients having relapses in substance abuse). Six clients (4.9%) completed all treatment sessions but did not provide data at all timepoints. A total of 32 (26.0%) clients completed all eight sessions of therapy and the post‐therapy measures and therefore had their data included in this study.

### Baseline demographic and clinical data

Demographic and baseline clinical data for all participants is shown in Table 1. There were no significant differences between the sample included in the analysis (*n* = 32) and all referrals who commenced CBT (*n* = 79) in the clinic during the period between July 2019 and August 2021.

**TABLE 1:** Baseline demographic and clinical data

	Study sample (*n* = 32)	All participants who commenced CBT (*n* = 79)
Age (*M* [*SD*], *n* = 29)	33.93 (16.16)	42.47 (23.77)
Length of time hearing voices (*M* [*SD*])	12.39 (11.17)	19.62 (25.78)
Gender, *n* (%)		
Female	18.00 (56.30)	42.00 (53.16)
Male	14.00 (43.80)	37.00 (46.84)
Highest level of education, *n* (%)		
Left school before 16	5.00 (15.60)	10.00 (12.80)
Left school at 16	4.00 (12.50)	13.00 (16.70)
Completing/ completed year 12	8.00 (25.00)	21.00 (26.90)
Completing/completed further vocational qualification	9.00 (28.10)	20.00 (25.60)
Completing/completed undergraduate degree	5.00 (15.60)	11.00 (14.10)
Completing/ completed postgraduate degree	1.00 (3.10)	3.00 (3.90)
Missing	0.00 (0.00)	1.00 (1.27)
Employment status, *n* (%)		
Unemployed	19.00 (59.40)	47.00 (59.50)
Employed (part or fulltime)	6.00 (18.80)	20.00 (25.30)
Student	7.00 (21.90)	12.00 (15.20)
Marital status, *n* (%)		
Single	20.00 (62.50)	56.00 (70.90)
Married/cohabiting/long term relationship	9.00 (12.50)	17.00 (21.50)
Separated/ divorced	3.00 (9.40)	6.00 (7.60)
Diagnosis on referral letter, *n* (%)		
Psychotic disorder	12.00 (37.50)	29.00 (32.90)
Multiple, including psychotic disorder	4.00 (12.50)	9.00 (19.00)
Non‐psychotic disorder	3.00 (9.40)	7.00 (5.10)
Multiple, no psychotic disorder	5.00 (15.60)	13.00 (19.00)
No diagnosis	0.00 (0.00)	1.00 (1.27)
Missing	8.00 (25.00)	20.00 (25.32)
Current medication, *n* (%)		
Anti‐psychotic medication only	7.00 (21.90)	21.00 (26.60)
Other psychotropic medication only	5.00 (15.60)	8.00 (10.10)
Combination (anti‐psychotic and other)	15.00 (46.90)	34.00 (43.50)
Missing	5.00 (15.60)	16.00 (20.25)

Abbreviations: *M*, mean; *n*, number; *SD*, standard deviation.

At baseline, participants had a median score of six (IQR 3.75) on the negative content subscale (highest possible score eight), suggesting that participants often heard voices that they perceived to be very negative in content. The median baseline score for the positive content scale was one (IQR 2), highest possible score four), suggesting that participants were not often hearing positive voices, but that positive voices were certainly part of many participants' experience.

While data relating to the exact content of voices was not routinely collected, common examples of negative voice content included threats or warnings of harm to the client or their loved ones, derogatory comments about the client's appearance, personality or life situation, and ego‐dystonic socially ‘taboo’ comments (e.g. sexually explicit, racist, or homophobic content).

### Changes in negative and positive content

Table 2. shows the median and interquartile range for negative and positive content of voices at each timepoint.

**TABLE 2:** Average scores on voices outcome measures at pre, mid and post‐treatment

	Pre treatment	Mid treatment	Post‐treatment
Negative voice content (Mdn, IQR)	6.00 (3.75)	6.00 (4.00)	4.00 (5.00)
Positive voice content (Mdn, IQR)	1.00 (2.00)	1.00 (2.00)	1.00 (1.00)
Voice severity (*M*, *SD*)	15.52 (3.50)	13.74 (4.77)	13.06 (4.34)
Voice‐related distress (Mdn, IQR)	3.00 (2.00)	2.00 (2.00)	2.00 (2.00)

Abbreviations: IQR, interquartile range; *M*, mean; Mdn, Median; *SD*, standard deviation.

A Friedman's test showed that there was a small to moderate, significant difference in negative voice content over the three timepoints, *X* ^*2*^_*F*_(2) = 17.58, *p* < .001, *W* = 0.28. Post‐hoc tests using the Dunn‐Bonferroni method showed that: (1) there was no significant difference between pretreatment and mid treatment (measuring the impact of the behavioural module of CBT, CSE), z = −1.44, *p* = .45; (2) negative voice content was found to be significantly lower at post‐treatment than at mid treatment (measuring the impact of the cognitive module of CBT), z = 3.81, *p* ≤ .001; and (3) there was no significant difference between pretreatment and post‐treatment (measuring the impact of the whole CBT intervention), z = 2.38, *p* = .53.

A Friedman's test showed that there was not a significant difference between positive voice content over the three timepoints, *X* ^*2*^_*F*_(2) = 4.03, *p* = .13, *W* = 0.06.

### Changes in voice‐related distress and voice severity

A Friedman's test showed that there was a small, significant difference in voice‐related distress over the three timepoints, X^2^F(2) = 10.76, *p* < .005, W = 0.17. Pairwise comparisons showed that voice‐related distress reduced significantly between pretreatment and mid‐treatment (*z* = 2.35, *p* = .02); (2) there was no significant change in voice‐related distress between mid‐treatment and post‐treatment (*z* = 0.25, *p* = .78); and (3) voice‐related distress reduced significantly overall (between pretreatment and post‐treatment; *z* = 2.60, *p* < .01).

A one‐way repeated ANOVA showed that there was a large, significant difference in voice severity over the three timepoints, *F* (2, 60) = 8.27, *p* < .001, partial η^2^ = .22. Pairwise comparisons showed that voice severity reduced significantly between pretreatment and mid‐treatment (*p* = .03); (2) there was no significant change in voice severity between mid‐treatment and post‐treatment (*p* = .34); and (3) voice severity reduced significantly overall (between pretreatment and post‐treatment; *p* < .01).

## DISCUSSION

The current study is the first to specifically examine changes in positive and negative voice content during CBT for distressing voices in a transdiagnostic sample, in a ‘real‐world’ clinical setting.

Before starting CBT for distressing voices, clients in an outpatient psychological therapy service reported often hearing voices that were very negative in content. The high levels of perceived negative content in this group of distressed voice hearers is in keeping with previous phenomenological research, which found that 60% of voice hearers in a psychiatric population endorsed hearing negative voice content (McCarthy‐Jones et al., 2014). Given that participants in the current study were specifically seeking treatment for distressing voices, and that distress (Rosen et al., 2018) and help seeking (Daalman et al., 2011; Honig et al., 1998) have been shown to be related to negative voice content, the high levels of negative content at baseline are not surprising. Of note, a number of clients also reported sometimes hearing voices with positive content. Indeed, this finding corroborates previous research that has shown that clinical voice hearers do report positive content that can co‐occur with negative voice content (McCarthy‐Jones et al., 2014).

Analyses indicated that our modular CBT intervention was associated with significant changes in the routinely reported metrics of voice‐related distress and overall voice severity. Both voice‐related distress and voice severity reduced significantly over the treatment, with the majority of change appearing to occur early in treatment (during the CSE phase, rather than during the cognitive therapy phase).

When examining omnibus tests of effects on voice content specifically, our findings showed that clients receiving a modular CBT for voices that has a focus on coping and on changing unhelpful beliefs about the self and voices had a significant reduction in negative voice content. This suggests that CBT for distressing voices may be associated with a small, but significant, reduction in negative voice content. Conversely, there was no significant change in positive voice content, suggesting that CBT for voices may not have an impact on positive voice content.

Post‐hoc analyses indicated that there was no significant change in negative content from pre to post‐therapy, but that significant change did occur in the latter half of therapy (from mid to post‐treatment) in which the cognitive therapy component was delivered. This may indicate that cognitive therapy is particularly associated with changes in negative content over and above coping focused therapy; however, it is not possible to determine whether this effect was truly down to the module content, or rather is due to a lagged or cumulative treatment effect. Notably, our analysis of effects of the treatment on voice‐related distress and voice severity differed, indicating that most change for these outcomes occurred in the early stages of therapy (during the coping focused phase). This discrepancy might indicate that while CSE is effective for reducing voice‐related distress and overall voice severity, it is less potent for addressing negative voice content specifically.

Although the study design limits conclusions about the active ingredients of the intervention that may have contributed to changes in negative voice content, the apparent response in the latter half of therapy does raise the possibility that the cognitive component of the intervention may be particularly effective in addressing negative voice content. The cognitive module of the treatment had a focus on two key areas of unhelpful beliefs: beliefs about the self and beliefs about voices. The proposal that changes in negative content occurred as a result of cognitive therapy for negative self‐schemas would corroborate previous research that has found a relationship between negative self‐schemas and higher levels of negative voice content (Smith et al., 2006; Thomas et al., 2015; Scott et al., 2020; for review on social schemata and voices see Paulik, 2012). The proposition that cognitive therapy addressing negative beliefs about voices led to a decrease in negative voice content is also in line with previous theory and research that has identified a likely bidirectional relationship between beliefs about voices and negative voice content. It has been identified that it is likely that negative voice content provides evidence for beliefs about the malevolence and power of the voice (Chadwick & Birchwood, 1994) and also that these beliefs regarding the intent and power of the voice then impact whether voice content is experienced as negative (e.g. in the case where objectively voice content is a neutral comment, but is interpreted to be negative due to wider beliefs about the intent of the voice; Larøi et al., 2019). It has also been suggested that beliefs about voice malevolence and omnipotence may lead to more negative interactions with voices (through hostile dialogue), which reinforce negative voice content through the strengthening of cognitive structures that are associated with negative content (Thomas et al., 2015). Our findings here therefore strengthen the proposition that negative schematic beliefs about self, others and voices play a role in negative voice content and also suggest that interventions targeting these beliefs (such as relating therapy and compassion‐focused therapy) are a fruitful direction for developing interventions that can specifically target negative voice content in situations in which this is the goal of the client.

The reductions in negative voice content from mid to post‐therapy were statistically significant but the effect size was small to moderate. This suggests that there is a need to develop interventions that have a more potent effect on negative voice content (given that negative voice content may play a key role in voice‐related distress and that change in negative voice content is a goal for many clients who seek support with their voices). Improving our understanding of the psychological processes that impact on negative voice content will be central to developing more effective interventions. It is plausible that different types of negative voice content are linked to different psychological mechanisms. A first step may therefore be to examine and categorize negative voice content in a more nuanced way. For example, differentiating ego‐dystonic socially taboo content that may be related to obsessive compulsive processes from derogatory content potentially related to negative self‐schema, and threatening content that might be conceptualized as relating to memories and social schema developed as a result of past experiences of interpersonal victimization and abuse (Larøi et al., 2019). Gathering empirical data in this area may then inform the development of targeted interventions that address these processes. Interventions such as compassion‐focused therapy or trauma focused therapies could be promising interventions where trauma memories or hypervigilance to threat are implicated (e.g. Brand et al., 2021; Heriot‐Maitland et al., 2019; Paulik, Hayward, et al., 2019; Paulik, Steel, & Arntz, 2019). Similarly, relationally based therapies, such as relating therapy (Hayward et al., 2017; Paulik et al., 2013), the Talking With Voices approach (e.g. Longden et al., 2021), or AVATAR therapy (Craig et al., 2018; Ward et al., 2020) may be indicated where relationships with voices mirror past experiences of discrimination, victimization, or marginalization and this appears to fuel negative content. Large trials that assess negative voice content as an outcome and which use mediation analysis to assess mechanisms of change in therapy would provide increased knowledge in this area.

The fact that our results indicated differential effects of CBT for voices on negative versus positive voice content highlights the fact that positive and negative content are likely not diametrically opposed concepts at ends of the same spectrum, but that perhaps they need to be considered as separate constructs that have different psychological mechanisms underpinning them. An important direction for future research may be in understanding the particular psychological factors involved in positive voice content and using this to develop interventions that might lead to increases in the amount of voice content that is perceived to be positive. This is an area that has been neglected in literature and interventions to date.

The use of real‐world data from clinical practice is a strength of this study, providing good ecological validity. However, a number of limitations must also be considered when interpreting the results found. Firstly, the study is an uncontrolled pre–post study, which limits the ability to ascribe the changes seen to the specific treatment offered, rather than to natural change over time, or to the general effects of a therapeutic relationship. Non‐random allocation to treatment and exclusion of those who did not complete treatment may have introduced some bias in the findings. The final sample was small and only a small proportion of those initially referred, this may have limited the power to detect treatment effects, as well as introducing bias to the outcomes. Future randomized controlled studies of psychological treatments for distressing voices could include negative voice content as an outcome of interest in order to provide more robust findings regarding the specific effects of these therapies on negative voice content. A commonly used outcome measure in trials of psychological interventions for voices has been the psychotic symptom rating scales (PSYRATS; Haddock et al., 1999), which does include an item relating to voice content, however as noted earlier, these trials have typically focused on voice‐related distress, voice frequency, or beliefs about voices as primary outcome measures, with the negative content item included as part of the voice‐related distress dimension. Increasingly, it is becoming clear that negative voice content may be a key variable of interest (Larøi et al., 2019). In addition, no follow‐up was conducted after the immediate post‐treatment timepoint so it is unclear if changes in negative voice content endure over longer timeframes.

Finally, the measures used to characterize negative and positive voice content in this study were limited to one or two item Likert scales, which are likely to be limited in capturing the complex and multidimensional nature of negative and positive voice content. Indeed, complexities in defining negative and positive voice content have been noted, with a recognition that perceptions of negative or positive content are likely to be influenced by objective voice content, as well as non‐verbal aspects (such as tone) and the person's beliefs about their voices, including the identity and intent of the voice (Badcock & Chhabra, 2013; Larøi et al., 2019). The measure in the current study measured subjective perceptions of voice content, but did not provide a decontextualized assessment of the literal content of voices. Future research could focus on developing a more comprehensive assessment tool for capturing relevant aspects of negative and positive voice content, this may be a multi‐item scale measuring different aspects of negative content (e.g. capturing both literal content and perceptions of communicative intent), or using qualitative methods.

In conclusion, the current findings suggest that current CBT treatments for distressing voices (that focus on increasing coping and addressing negative beliefs about voices) may be associated with reductions in negative, but not positive, voice content. There is a need to enhance these effects by developing treatments that target specific processes involved in negative and positive voice content and to further explore efficacy in well‐powered, controlled trials with more comprehensive measures of voice content.

## CONFLICTS OF INTEREST

The authors have no conflicts of interest to declare.

## AUTHOR CONTRIBUTIONS

**Rachel M. Brand:** Conceptualization; Formal analysis; Writing – original draft; Writing – review & editing. **Johanna C. Badcock:** Conceptualization; Methodology; Writing – review & editing. **Georgie Paulik:** Conceptualization; Data curation; Methodology; Project administration; Supervision; Writing – review & editing.

## Data Availability

The data that support the findings of this study are available from the corresponding author upon reasonable request.

## References

[papt12399-bib-0001] Badcock, J. C. , & Chhabra, S. (2013). Voices to reckon with: Perceptions of voice identity in clinical and non‐clinical voice hearers. Frontiers in Human Neuroscience, 7, 114. 10.3389/fnhum.2013.00114 23565088 PMC3615181

[papt12399-bib-0002] Badcock, J. C. , Graham, M. E. , & Paulik, G. (2020). Assessing the clinical significance of treatment outcomes for distressing voices in routine clinical practice. Clinical Psychology and Psychotherapy., 27, 79–86. 10.1002/cpp.2410 31659810

[papt12399-bib-0003] Begemann, M. , Sommer, I. E. , Brand, R. M. , Oomen, P. P. , Jongeneel, A. , Berkhout, J. , Molenaar, R. E. , Wielage, N. N. , Toh, W. L. , Rossell, S. L. , & Bell, I. H. (2021). Auditory verbal hallucinations and childhood trauma subtypes across the psychosis continuum: A cluster analysis. Cognitive Neuropsychiatry, 27(2‐3), 150–168. 10.1080/13546805.2021.1925235 33980128

[papt12399-bib-0004] Berry, C. , Newcombe, H. , Strauss, C. , Rammou, A. , Schlier, B. , Lincoln, T. , & Hayward, M. (2021). Validation of the Hamilton program for schizophrenia voices questionnaire: Associations with emotional distress and wellbeing, and invariance across diagnosis and sex. Schizophrenia Research, 228, 336–343. 10.1016/j.schres.2020.12.032 33540145

[papt12399-bib-0005] Berry, K. , Wearden, A. , Barrowclough, C. , Oakland, L. , & Bradley, J. (2012). An investigation of adult attachment and the nature of relationships with voices. The British Journal of Clinical Psychology, 51(3), 280–291. 10.1111/j.2044-8260.2011.02027.x 22803935

[papt12399-bib-0006] Birchwood, M. , Michail, M. , Meaden, A. , Tarrier, N. , Lewis, S. , Wykes, T. , Davies, L. , Dunn, G. , & Peters, E. (2014). Cognitive behaviour therapy to prevent harmful compliance with COMMAND hallucinations (COMMAND): A randomised controlled trial. The Lancet Psychiatry, 1(1), 23–33. 26360400 10.1016/S2215-0366(14)70247-0

[papt12399-bib-0007] Brand, R. M. , Bendall, S. , Hardy, A. , Rossell, S. L. , & Thomas, N. (2021). Trauma‐focused imaginal exposure for auditory hallucinations: A case series. Psychology and Psychotherapy, 94 Suppl 2(Suppl 2), 408–425. 10.1111/papt.12284 32436342 PMC8246845

[papt12399-bib-0045] Chadwick, P. , & Birchwood, M. (1994). The omnipotence of voices: A cognitive approach to auditory hallucinations. The British Journal of Psychiatry, 164, 190–201. https://psycnet.apa.org/record/1994‐34382‐001 8173822 10.1192/bjp.164.2.190

[papt12399-bib-0008] Corstens, D. , & Longden, E. (2013). The origins of voices: Links between life history and voice hearing in a survey of 100 cases. Psychosis, 5(3), 270–285. 10.1080/17522439.2013.816337

[papt12399-bib-0009] Craig, T. K. J. , Rus‐Calafell, M. , Ward, T. , Leff, J. P. , Huckvale, M. , Howarth, E. , Emsley, R. , & Garety, P. A. (2018). AVATAR therapy for auditory verbal hallucinations in people with psychosis: A single‐blind, randomised controlled trial. The Lancet Psychiatry, 5(1), 31–40. 10.1016/S2215-0366(17)30497-2 29175276 PMC5746597

[papt12399-bib-0010] Daalman, K. , Boks, M. P. , Diederen, K. M. , de Weijer, A. D. , Blom, J. D. , Kahn, R. S. , & Sommer, I. E. (2011). The same or different? A phenomenological comparison of auditory verbal hallucinations in healthy and psychotic individuals. The Journal of Clinical Psychiatry, 72(3), 320–325. 10.4088/JCP.09m05797yel 21450152

[papt12399-bib-0044] Deamer, F. , & Wilkinson, S. (2015). The speaker behind the voice: Therapeutic practice from the perspective of pragmatic theory. Frontiers in Psychology, 6, 10.3389/fpsyg.2015.00817 PMC4463863 26124738

[papt12399-bib-0011] de Boer, J. N. , Corona Hernández, H. , Gerritse, F. , Brederoo, S. G. , Wijnen, F. , & Sommer, I. E. (2021). Negative content in auditory verbal hallucinations: A natural language processing approach. Cognitive Neuropsychiatry, 27(2‐3), 139–149. 10.1080/13546805.2021.1941831 34154512

[papt12399-bib-0012] Garwood, L. , Dodgson, G. , Bruce, V. , & McCarthy‐Jones, S. (2015). A preliminary investigation into the existence of a hypervigilance subtype of auditory hallucination in people with psychosis. Behavioural and Cognitive Psychotherapy, 43(01), 52–62. 10.1017/S1352465813000714 23962410

[papt12399-bib-0013] Haddock, G. , McCarron, J. , Tarrier, N. , & Faragher, E. B. (1999). Scales to measure dimensions of hallucinations and delusions: The psychotic symptom rating scales (PSYRATS). Psychological Medicine, 29(4), 879–889. 10.1017/s0033291799008661 10473315

[papt12399-bib-0014] Hardy, A. , Fowler, D. , Freeman, D. , Smith, B. , Steel, C. , Evans, J. , Garety, P. , Kuipers, E. , Bebbington, P. , & Dunn, G. (2005). Trauma and hallucinatory experience in psychosis. The Journal of Nervous and Mental Disease, 193(8), 501–507. 10.1097/01.nmd.0000172480.56308.21 16082293

[papt12399-bib-0015] Hayward, M. , Strauss, C. , & Kingdon, D. (2012). Overcoming distressing voices. Constable & Robinsons Ltd.

[papt12399-bib-0016] Hayward, M. , Jones, A. M. , Bogen‐Johnston, L. , Thomas, N. , & Strauss, C. (2017). Relating therapy for distressing auditory hallucinations: A pilot randomized controlled trial. Schizophrenia Research, 183, 137–142. 10.1016/j.schres.2016.11.019 27916286

[papt12399-bib-0017] Hazell, C. , Hayward, M. , Cavanagh, K. , Jones, A.‐M. , & Strauss, C. (2018). Guided self‐help cognitive‐behaviour intervention for VoicEs (GiVE): Results from a pilot randomised controlled trial in a transdiagnostic sample. Schizophrenia Research, 195, 441–447. 10.1016/j.schres.2017.10.004 29033279

[papt12399-bib-0018] Heriot‐Maitland, C. , McCarthy‐Jones, S. , Longden, E. , & Gilbert, P. (2019). Compassion focused approaches to working with distressing voices. Frontiers in Psychology, 10, 152. 10.3389/fpsyg.2019.00152 30774614 PMC6367219

[papt12399-bib-0019] Honig, A. , Romme, M. A. , Ensink, B. J. , Escher, S. D. , Pennings, M. H. , & deVries, M. W. (1998). Auditory hallucinations: A comparison between patients and nonpatients. The Journal of Nervous and Mental Disease, 186(10), 646–651. 10.1097/00005053-199810000-00009 9788642

[papt12399-bib-0020] Jamieson, S. (2004). Likert scales: How to (ab)use them. Medical Education, 38(12), 1217–1218. 10.1111/j.1365-2929.2004.02012.x 15566531

[papt12399-bib-0021] Johns, L. C. , Kompus, K. , Connell, M. , Humpston, C. , Lincoln, T. M. , Longden, E. , Preti, A. , Alderson‐Day, B. , Badcock, J. C. , Cella, M. , Fernyhough, C. , McCarthy‐Jones, S. , Peters, E. , Raballo, A. , Scott, J. , Siddi, S. , Sommer, I. E. , & Larøi, F. (2014). Auditory verbal hallucinations in persons with and without a need for care. Schizophrenia Bulletin, 40(Suppl 4), S255–S264. 10.1093/schbul/sbu005 24936085 PMC4141313

[papt12399-bib-0022] Larøi, F. , Thomas, N. , Aleman, A. , Fernyhough, C. , Wilkinson, S. , Deamer, F. , & McCarthy‐Jones, S. (2019). The ice in voices: Understanding negative content in auditory‐verbal hallucinations. Clinical Psychology Review, 67, 1–10. 10.1016/j.cpr.2018.11.001 30553563

[papt12399-bib-0023] Loberg, E. M. , Gjestad, R. , Posserud, M. B. , Kompus, K. , & Lundervold, A. J. (2019). Psychosocial characteristics differentiate non‐distressing and distressing voices in 10,346 adolescents. European Child and Adolescent Psychiatry, 28(10), 1353–1363. 10.1007/s00787-019-01292-x 30820670 PMC6785583

[papt12399-bib-0024] Longden, E. , Corstens, D. , Morrison, A. P. , Larkin, A. , Murphy, E. , Holden, N. , Steele, A. , Branitsky, A. , & Bowe, S. (2021). A treatment protocol to guide the delivery of dialogical engagement with auditory hallucinations: Experience from the talking with voices pilot trial. Psychology and Psychotherapy, 94(3), 558–572. 10.1111/papt.12331 33629816

[papt12399-bib-0025] McCarthy‐Jones, S. , Trauer, T. , Mackinnon, A. , Sims, E. , Thomas, N. , & Copolov, D. L. (2014). A new phenomenological survey of auditory hallucinations: Evidence for subtypes and implications for theory and practice. Schizophrenia Bulletin, 40(1), 231–235. 10.1093/schbul/sbs156 23267192 PMC3885292

[papt12399-bib-0026] Nayani, T. , & David, A. (1996). The auditory hallucination: A phenomenological survey. Psychological Medicine, 26(1), 177–189. 10.1017/S003329170003381X 8643757

[papt12399-bib-0027] Paulik, G. (2012). The role of social schema in the experience of auditory hallucinations: A systematic review and a proposal for the inclusion of social schema in a cognitive behavioural model of voice hearing. Clinical Psychology and Psychotherapy, 19(6), 459–472. 10.1002/cpp.768 21774037

[papt12399-bib-0028] Paulik, G. , Hayward, M. , & Birchwood, M. (2013). Cognitive Behavioural relating therapy (CBRT) for voice hearers: A case study. Behavioural and Cognitive Psychotherapy, 41(5), 626–631. 10.1017/S1352465812001014 23211878

[papt12399-bib-0029] Paulik, G. , Hayward, M. , Jones, A.‐M. , & Badcock, J. C. (2019). Evaluating the “C” and “B” in brief cognitive behaviour therapy for distressing voices in routine clinical practice in an uncontrolled study. Clinical Psychology and Psychotherapy, 26, 734–742. 10.1002/cpp.2395 31472014

[papt12399-bib-0030] Paulik, G. , Steel, C. , & Arntz, A. (2019). Imagery rescripting for the treatment of trauma in voice hearers: A case series. Behavioural and Cognitive Psychotherapy, 47(6), 709–725. 10.1017/S1352465819000237 30975230

[papt12399-bib-0043] Peters, E. R. , Williams, S. L. , Cooke, M. A. , & Kuipers, E. (2012). It's not what you hear, it's the way you think about it: Appraisals as determinants of affect and behaviour in voice hearers. Psychological Medicine, 42(7), 1507–1514. 10.1017/s0033291711002650 22115329

[papt12399-bib-0031] Rosen, C. , McCarthy‐Jones, S. , Jones, N. , Chase, K. A. , & Sharma, R. P. (2018). Negative voice‐content as a full mediator of a relation between childhood adversity and distress ensuing from hearing voices. Schizophrenia Research, 199, 361–366. 10.1016/j.schres.2018.03.030 29580740 PMC6151289

[papt12399-bib-0032] Scott, M. , Rossell, S. L. , Meyer, D. , Toh, W. L. , & Thomas, N. (2020). Childhood trauma, attachment and negative schemas in relation to negative auditory verbal hallucination (AVH) content. Psychiatry Research, 290, 112997. 10.1016/j.psychres.2020.112997 32470717

[papt12399-bib-0033] Smith, B. , Fowler, D. G. , Freeman, D. , Bebbington, P. , Bashforth, H. , Garety, P. , Dunn, G. , & Kuipers, E. (2006). Emotion and psychosis: Links between depression, self‐esteem, negative schematic beliefs and delusions and hallucinations. Schizophrenia Research, 86(1–3), 181–188. 10.1016/j.schres.2006.06.018 16857346

[papt12399-bib-0034] Tarrier, N. , Beckett, R. , Harwood, S. , Baker, A. , Yusupoff, L. , & Ugarteburu, I. (1993). A trial of two cognitive‐behavioural methods of treating drug‐ resistant residual psychotic symptoms in schizophrenic clients: I Outcome. British Journal of Psychiatry, 162, 524–532. 10.1192/bjp.162.4.524 8481745

[papt12399-bib-0035] Tarrier, N. , Yusupoff, L. , Kinney, C. , McCarthy, E. , Gledhill, A. , Haddock, G. , & Morris, J. (1998). Randomised controlled trial of intensive cognitive behaviour therapy with chronic schizophrenia. British Medical Journal, 317, 303–307. 10.1136/bmj.317.7154.303 9685273 PMC28621

[papt12399-bib-0036] Thomas, N. , Farhall, J. , & Shawyer, F. (2015). Beliefs about voices and schemas about self and others in psychosis. Behavioural and Cognitive Psychotherapy, 43(2), 209–223. 10.1017/S1352465813000817 24103156

[papt12399-bib-0037] van der Gaag, M. , Hageman, M. , & Birchwood, M. (2003). Evidence for a cognitive model of auditory hallucinations. The Journal of Nervous and Mental Disease, 191(8), 542–545. 10.1097/01.nmd.0000082183.95853.ec 12972858

[papt12399-bib-0038] van der Gaag, M. , Valmaggia, L. R. , & Smit, F. (2014). The effects of individually tailored formulation‐based cognitive behavioural therapy in auditory hallucinations and delusions: A meta‐analysis. Schizophrenia Research, 156(1), 30–37. 10.1016/j.schres.2014.03.016 24731619

[papt12399-bib-0039] van Lieshout, R. J. , & Goldberg, J. O. (2007). Quantifying self‐reports of auditory verbal hallucinations in persons with psychosis. Canadian Journal of Behavioural Science, 39, 73–77. 10.1037/cjbs2007006

[papt12399-bib-0042] Valavanis, S. , Thompson, C. , & Murray, C. D. (2019). Positive aspects of voice‐hearing: A qualitative metasynthesis. Mental Health, Religion & Culture, 22(2), 208–225. 10.1080/13674676.2019.1601171

[papt12399-bib-0040] Ward, T. , Rus‐Calafell, M. , Ramadhan, Z. , Soumelidou, O. , Fornells‐Ambrojo, M. , Garety, P. , & Craig, T. (2020). AVATAR therapy for distressing voices: A comprehensive account of therapeutic targets. Schizophrenia Bulletin, 46(5), 1038–1044. 10.1093/schbul/sbaa061 PMC7505185 32372082

[papt12399-bib-0041] Waters, F. , & Fernyhough, C. (2017). Hallucinations: A systematic review of points of similarity and difference across diagnostic classes. Schizophrenia Bulletin, 43(1), 32–43. 10.1093/schbul/sbw132 27872259 PMC5216859

